# Investigating the presence of microplastics in demersal sharks of the North-East Atlantic

**DOI:** 10.1038/s41598-020-68680-1

**Published:** 2020-07-22

**Authors:** Kristian J. Parton, Brendan J. Godley, David Santillo, Muhammad Tausif, Lucy C. M. Omeyer, Tamara S. Galloway

**Affiliations:** 1 Centre for Ecology and Conservation, College of Life and Environmental Sciences, University of Exeter, Penryn Campus, Penryn, TR10 9EZ UK; 2 Greenpeace Research Laboratories, School of Biosciences, Innovation Centre Phase 2, University of Exeter, Exeter, UK; 3 Textile Technology Research Group, School of Design, University of Leeds, Leeds, LS2 9JT UK; 4 Biosciences, College of Life and Environmental Sciences, Geoffrey Pope Building, University of Exeter, Stocker Road, Exeter, Devon EX4 4QD UK

**Keywords:** Marine biology, Ocean sciences, Ichthyology, Environmental impact

## Abstract

Microplastic pollution is ubiquitous in the marine environment and is ingested by numerous marine species. Sharks are an understudied group regarding their susceptibility to microplastic ingestion. Here, we provide evidence of ingestion of microplastic and other anthropogenic fibres in four demersal sharks species found in the waters of the United Kingdom and investigate whether body burdens of contamination vary according to species, sex or size. Sharks were collected from the North-East Atlantic. Stomachs and digestive tracts of 46 sharks of 4 species were examined and 67% of samples contained at least one contaminant particle. Although we acknowledge modest sample size, estimated particle burden increased with body size but did not vary systematically with sex or species. A total of 379 particles were identified, leading to median estimates ranging from 2 to 7.5 ingested contaminants per animal for the 4 species. The majority were fibrous in nature (95%) and blue (88%) or black (9%) in colour. A subsample of contaminants (N = 62) were subject to FT-IR spectroscopy and polymers identified as: synthetic cellulose (33.3%), polypropylene (25%), polyacrylamides (10%) and polyester (8.3%). The level of risk posed to shark species by this level of contamination is unknown. Nevertheless, this study presents the first empirical evidence and an important baseline for ingestion of microplastics and other anthropogenic fibres in native UK shark species and highlights the pervasive nature of these pollutants.

## Introduction

### Plastics in the marine environment

Research on plastic in the marine environment has accelerated rapidly in the last decade, with numerous publications describing its impact on ecosystems and marine taxa^1–7^. It is estimated that between 4.8 and 12.7 million tonnes of plastic enter the oceans every year from a variety of sources^6^. Plastic is a popular material due to its durability, low production cost and efficiency in its uses^8^. It is these properties, alongside its often disposable nature that leads to its prevalence in the environment for many years^9^.

Microplastics (defined as plastic particles < 5 mm)^10^ are ubiquitous in the marine environment^11–13^. Despite this knowledge, quantitative assessments of their abundance are still fairly limited^14^, although some estimates place their abundance at 5.25 trillion particles globally, weighing in at over 250,000 tonnes^5^. Microplastics, in the form of fibres, fragments or beads/spheres, assimilate in the marine ecosystem via multiple avenues. Larger pieces of plastic can disintegrate over time due to UV radiation exposure, wave action and physical abrasion, eventually fragmenting into microscopic particles^15^. Microplastics are also found in many everyday items used by humans including cosmetic products and can be produced by clothing wear^16–19^. These can then reach the oceans via wastewater treatment plants^20^.

### Ingestion of microplastics in marine species

Ingestion of microplastics is reported in many marine species including turtles, marine mammals and fish^1,21–25^. Alongside these larger species, microplastics have been reported in invertebrates such as zooplankton and crustaceans^26–28^. Our understanding of the impacts of microplastic ingestion is better understood in the latter group, with reports suggesting dose-dependent detrimental effects on feeding behaviour, development, reproduction and lifespan^29–31^.

### Microplastic ingestion in elasmobranchs

Elasmobranchs are relatively understudied in regards to threats from plastic pollution^32,33^, nonetheless their susceptibility to microplastic ingestion has been reported in a handful of scientific publications^22,34–39^. It is thought that some species of elasmobranch may be at higher risk of microplastic ingestion based on their feeding strategies or habitat use^35^. Filter feeding species (such as whale sharks and basking sharks) that occupy habitats which overlap areas with high densities of plastic pollution have been suggested to be at higher risk of microplastic ingestion^35,40,41^. Many shark species, however, are non-filter feeders, instead feeding on a range of larger organisms such as fish, crustaceans, marine turtles and marine mammals, all of which have records of microplastic ingestion^22–24,27^.

### North-East Atlantic demersal elasmobranchs

The North-East Atlantic is home to numerous shark and ray species, including small to medium sized demersal sharks. These species can be found at varying depths from 5 to 900 m^42,43^, most often residing in benthic habitats^44,45^. They feed on a wide range of small teleost fishes, crustaceans and cephalopods^44,46^. Due to their habitat choice they are often caught in demersal fisheries as bycatch, however targeted fisheries for these species also exist^47,48^. The exposure of microplastics to demersal shark species globally, is currently poorly investigated, with only a few reports of plastic ingestion, mostly situated in and around the Mediterranean Sea^22,36–38,49,50^. There have, however, been multiple studies of plastic ingestion in bony fish in the region, with ingestion rates varying from 1 to 47% across the species^22,51–54^.

Here we carry out the first detailed comparative study of microplastic ingestion in four shark species in the North-East Atlantic (small-spotted catshark; *Scyliorhinus canicula*, starry smooth-hound; *Mustelus asterias*, spiny dogfish; *Squalus acanthias* and bull huss; *Scyliorhinus stellaris*). These species were chosen due to their availability as bycatch in local fisheries. Alongside this, all four species are primarily demersal in their habitat choice, therefore studying microplastic ingestion within them may provide insights into contaminant levels for this marine biome and as a result indicate whether these species would be suitable bio-indicators for marine pollution. Given interspecific differences in habitat niche, ontogenetic shifts in diet and sex variation in life history strategies, we hypothesized that there would be differences in contaminant load among species, between sex and among size classes.

## Materials and methods

### Collection and dissection of shark samples

The study was conducted in Cornwall, UK using sharks caught as bycatch in a demersal hake fishery, fishing in and around the North-East Atlantic and Celtic Sea (ICES rectangles: VIIg, VIIh and VIIf). Four species of sharks were investigated (Total N = 46), including: small-spotted catshark (*Scyliorhinus canicula*) (n = 12), spiny dogfish (*Squalus acanthias*) (n = 12), starry smooth-hound (*Mustelus asterias)* (n = 12) and bull huss (*Scyliorhinus stellaris*) (n = 10). Standard shark morphometric measurements were taken for each species (for full details see Supplementary Materials).

### Necropsy and analysis

Upon dissection, the entire gastrointestinal tracts were removed (stomach and intestines) and 10 ml (20–50% of total volume depending on species) of their contents were removed for analysis and visual inspection of gut contents (see Supplementary Fig. S1). Samples were treated with 20% potassium hydroxide (KOH) as recent studies have highlighted its efficacy at digesting fish ingesta^53,55–57^ and heated for 48 h at 60 °C to aid digestion of biological materials. Digested samples were filtered and subsequently analysed under a digital stereo microscope (Leica M165C) and classified by type (fibre, fragment or bead) and colour, as well as measured (mm). A subsample of the contaminants identified (including potential fragments and fibres) underwent Fourier Transform Infrared spectroscopy (FT-IR) to gain insights into their polymer make-up and possible origins. Substantial measures were taken to reduce and control for contamination of samples throughout laboratory work, including the running of procedural blanks and air-borne contamination blanks at every stage of the necropsy and subsequent analysis (for full details, including quality control and contamination control measures see Supplementary Materials). All methods were carried out in accordance with relevant guidelines and regulations.

All statistical analyses were conducted on raw data. A negative binomial generalised linear model (GLM) was used to investigate the influence of species, sex and individual length on the estimated number of ingested fibres, using the MASS package^58^ in R v3.5.1.^59^ All combinations of terms were examined and ranked by Akaike’s Information Criteria (AIC) using subset selection of the maximal model using the MuMIn package v1.42.1.^60^ Top ranked models were defined as models ΔAIC ≤ 2 units of the best supported model, after excluding further models where a simpler model attained stronger weighting^61^.

### Particle terminology

Throughout the manuscript a range of terms are used to describe the various identified contaminants. The following terms are hereby explained. Microplastics and/or microplastic fibres refer to traditional petrochemical-derived polymer compounds. Anthropogenic fibres encompass compounds that are naturally occurring, however have been repurposed for human use, this includes the likes of synthetic regenerated cellulose, viscose, rayon and cotton. Contaminants/contaminant particles, in this context, refers to both microplastics and anthropogenic fibres as an umbrella term for compounds not-naturally occurring within these sharks.

## Results

### Descriptive statistics

In total, 46 individual sharks were analysed, of which 56.5% were male, although proportion varied across species (Proportion male for individual species: small-spotted catshark 66.6%, starry smooth-hound 25%, spiny dogfish 83.3%, bull huss 50%). Overall, 67.4% of sharks were classified as adults although again, the proportion differed among species (Proportion adult for individual species: small-spotted catshark 75%, starry smooth-hound 66.6%, spiny dogfish 58.3%, bull huss 58.3%).

Almost all particles identified in sharks were classified as fibres, with only two fragments identified, and no beads/spheres found. Of the 46 sharks analysed in this study, samples from 67% (31/46) contained at least one contaminant particle and incidence was relatively consistent across species (small-spotted catshark 66.6%, starry smooth-hound 75%, spiny dogfish 58%, bull huss 70%). Estimated number of fibres varied across the four shark species: estimated median fibres (IQ range; range): Overall: 4(0–9; 0–770) (IQ range; range), starry smooth-hound (7.5(3.8–28.75; 0–735), small spotted catshark (2(0–4; 0–6), spiny dogfish (4(0–4; 0–12), bull huss (5(1.3–13.8; 0–770).

**Figure 1:**
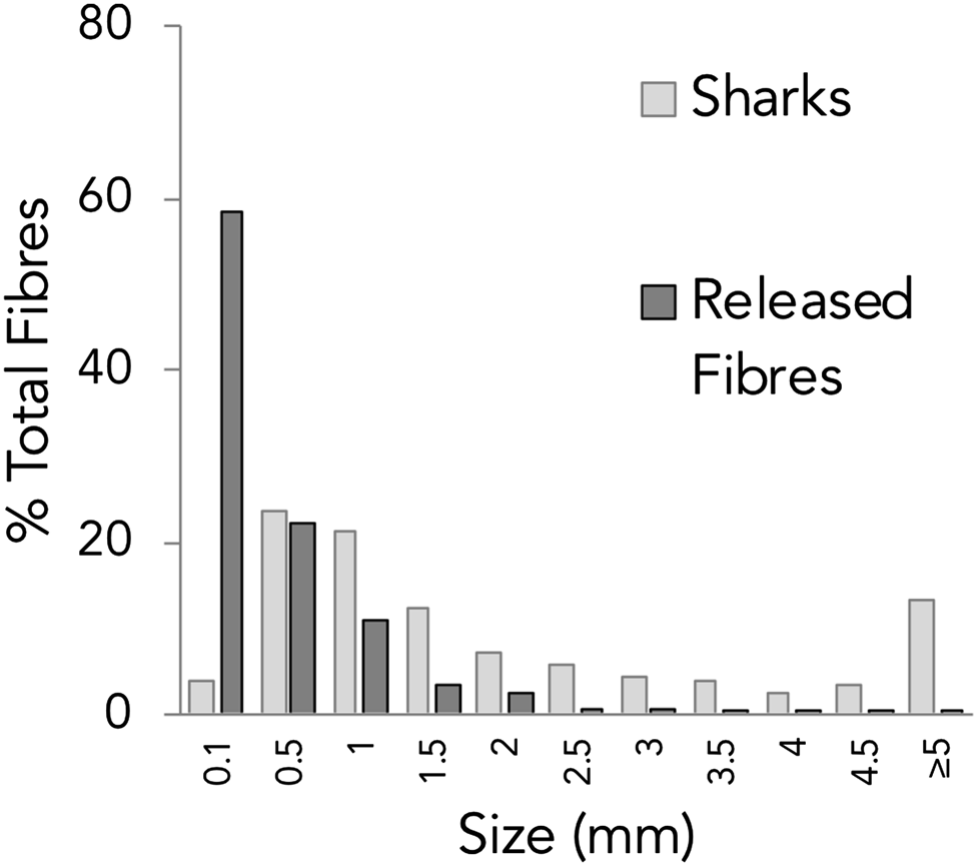
Fibre length distribution. Fibre lengths as a proportion of total fibres for fibres found in shark species (light grey) and fibres released in laboratory conditions after washing of various cotton and polyethylene terephthalate textiles. Palacios Marin AV, (2019) Release of microfibres from comparative common textile structures during laundering (Unpublished Masters dissertation). University of Leeds, UK.

**Figure 2:**
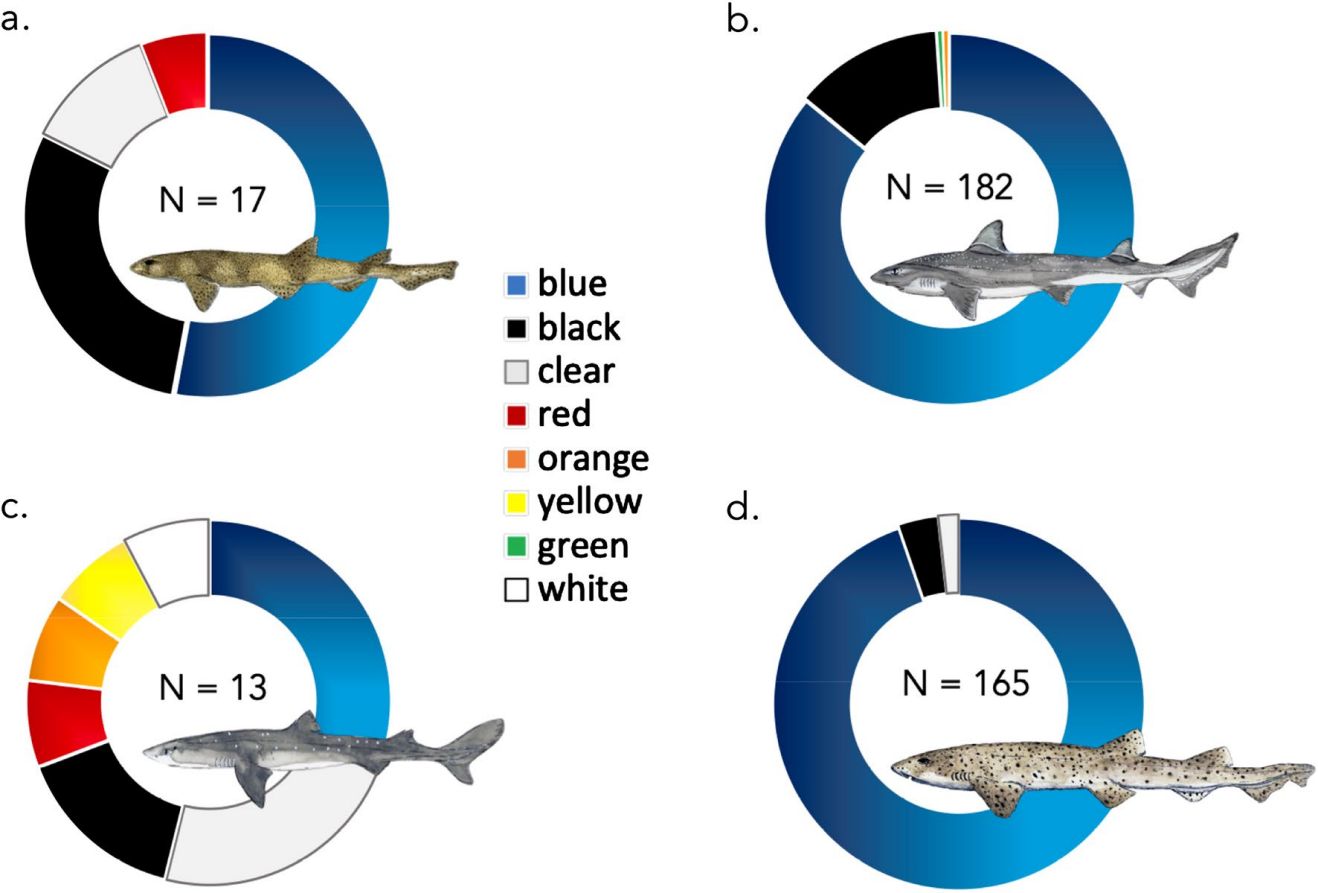
Composition of colours of ingested fibres, found across both the stomachs and intestines of four species of north-east Atlantic demersal sharks: (**a)** small-spotted catshark (*Scyliorhinus canicula*), (**b**) starry smooth-hound (*Mustelus asterias*), (**c**) spiny dogfish (*Squalus acanthias*) and (**d**) bull huss (*Scyliorhinus stellaris*). Total N of coloured fibres identified annotated within figure. Elasmobranch drawings by Lucie Jones.

Fibres ranged in length from 0.3 mm to 14.4 mm and had an average length of 2.7 mm ± 2.6 SD (see Fig. 1). The vast majority of fibres were blue (88.0%) or black (8.8%) in colour, with the remaining colours including: red, yellow and other (clear, green and white) each making up 3.8% (see Fig. 2 A-D). The two fragments identified were blue and white in colour. Fibres larger than 5 mm (n = 50) were considered here as macroplastics and were excluded from the analysis, although can be found grouped together in the ≥ 5 mm category on Fig. 1.

### Differences between species, sex and body size

**Figure 3:**
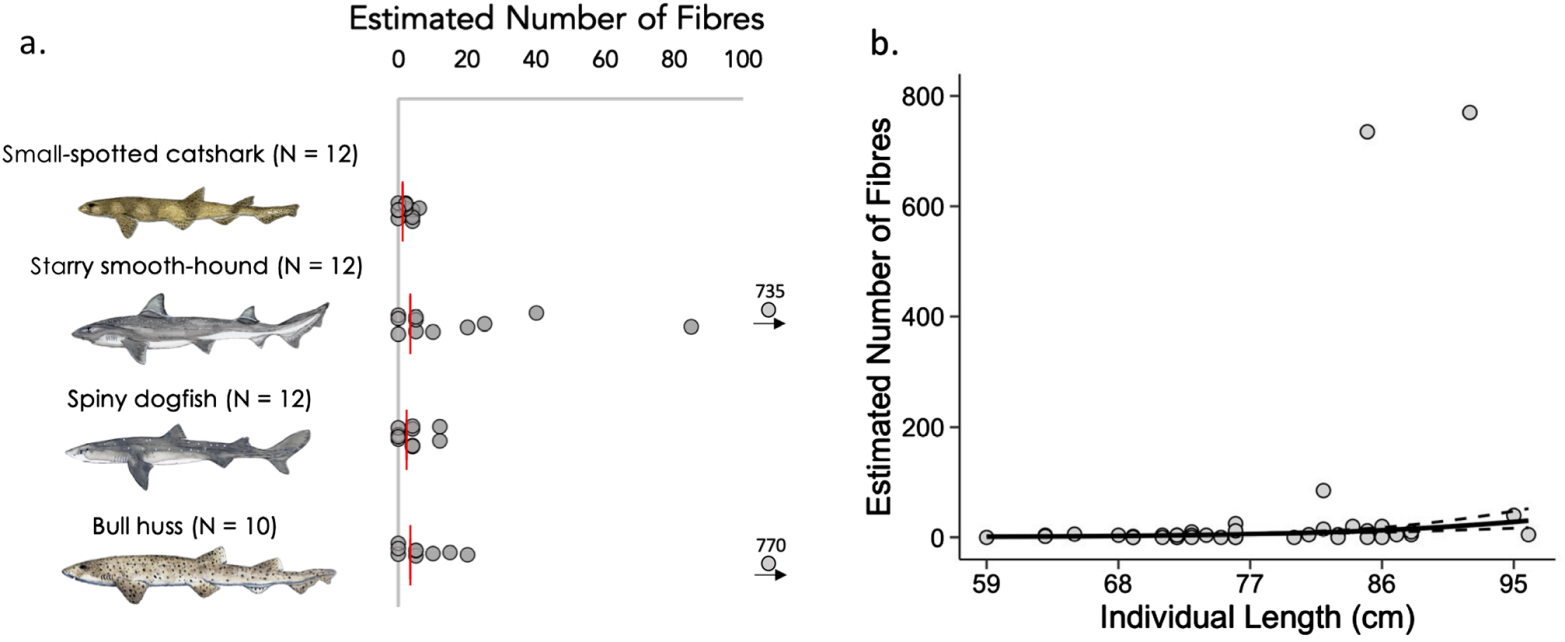
Estimated fibre ingestion and relationship with total length (cm). (**a**) Expected number of fibres based on extrapolation from full stomach/GI tract volumes. Medians marked by red line. N = annotated. Elasmobranch drawings by Lucie Jones. (**b**) Relationship between the estimated number of ingested fibres and individual length. Lines denote predictions from the top ranked model presented in Supplementary Table S2. Standard errors are shown by the dashed lines.

The estimated number of ingested microfibres was positively influenced by individual shark body length, however it did not differ between species or sex (See Fig. 3A,B, Supplementary Table S2 and Supplementary Fig. S5). It should be noted two individuals in this study (one starry smooth-hound and one bull huss) had much higher levels, with the sample from the former individual containing 147 fibres and the sample from the latter containing 154 fibres. Upon visual examination, these fibres appeared to be strands of blue rope, subsequently confirmed as olefin polypropylene. (Supplementary Figures S4–S8 have been created with these outliers removed/added for comparison).

### Polymer identification

A subsample of contaminant particles (n = 60 fibres, n = 2 fragments) were subject to FT-IR analysis (16% of total contaminants identified). However, when we consider the sample set without the two outliers mentioned above which were olefin polypropylene fibres, the subsample of contaminants that underwent FT-IR spectroscopy equalled 79% of all particles isolated.

Our analysis revealed 33.3% of fibres (n = 20) were cellulose derivatives (Alpha & Ecteola modified), however further analysis by light microscopy revealed these cellulose fibres were anthropogenic in nature due to their uniform diameter distribution across the fibre length and observation of convoluted structure of the fibre; a characteristic of cotton fibres (see Supplementary Fig. S2). Polyacrylamides made up 10% of fibres (n = 6), 8.3% of fibres were polyesters (n = 5) and 1.7% were cellophane (n = 1). Another 25% (n = 15) registered as Olefin polypropylene. Combined with the aforementioned microplastic contaminants (polyester and polyacrylamide), this results in a total of 43.3% of particles being true microplastics.

**Figure 4:**
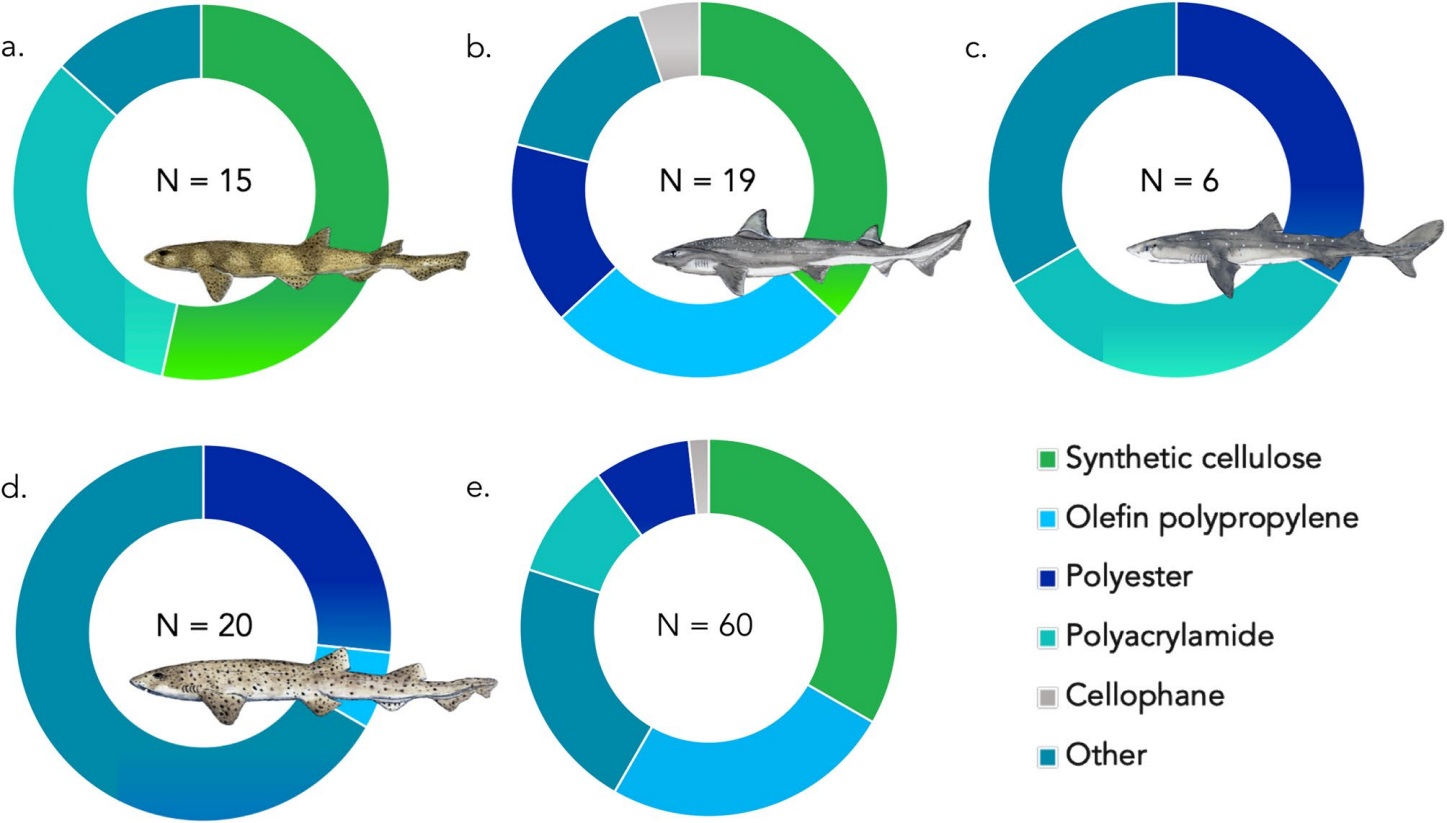
Composition of polymer make up of fibres between shark species. N of polymers identified in each species annotated on figure. (**a**) small-spotted catshark. (**b**) starry smooth-hound. (**c**) Spiny dogfish. (**d**) bull huss. (**e**) Total polymer percentages for all four species. Other = Biological materials and/or low spectral match scores. Elasmobranch drawings by Lucie Jones.

The remaining 21.6% of fibres (n = 13) were either unidentifiable due to low spectral match scores (n = 7) or returned as biological in nature (n = 6). Biological returns were excluded from broader statistical analysis. See Fig. 4 & Supplementary Table S1.

The two fragments identified returned as polyethylene and polypropylene (see Supplementary Fig. S3).

## Discussion

Our study is the first to demonstrate the presence of microplastic and anthropogenic particle contaminants in resident UK shark species in the North-East Atlantic. Despite there being no substantial differences in microplastic uptake among the shark species studied here, the research provides an important empirical baseline for future work investigating contaminant levels in UK sharks. Greater levels of contamination might be expected in animals that inhabit other parts of the UK with lower water quality. Although we have not demonstrated any health impacts on the sharks, the presence of these particle contaminants indicates their pervasiveness in the marine environment. With increasing global plastic production and its prevalence in every day products, the abundance of such marine pollutants is likely set to increase.

### Contaminant particle ingestion by species, sex and size

Nearly 70% of all sharks sampled in our study contained at least one contaminant particle in their digestive tracts. Although this is likely to be a conservative estimate of incidence, this number is significantly higher than many other reports for similar shark species around the world^22,36,39,50,62,63^ see Table 1. Studies by Alomar and Deudero^38^ and Smith^36^ revealed ingestion rates of microplastics at 16.8% in blackmouth catsharks sampled in the Mediterranean, and 15% in small-spotted catsharks from the North sea, respectively^36,63^. Interestingly, the Mediterranean is considered by some to be one of the worst affected oceans with regards to plastic pollution^5,64,65^, therefore ingestion of contaminant particles may have been expected to have been lower in North-East Atlantic. The only other study to have been conducted on similar species and within a similar ocean area is that of Neves et al.^22^, which found microplastic ingestion rates of 20% in small-spotted catsharks collected from the North-East Atlantic coast of Portugal, with microplastics being mostly fibrous in nature.

**Table 1:** Breakdown of publications on elasmobranchs and microplastics, featuring ocean basin, location, species examined, Number of samples examined, methodology for extraction and percentage of contaminant ingestion for species studied.

Ocean basin	Species examined	N	Methodology	% Ingestion	References
**Atlantic**					
North-East	*Scyliorhinus canicula*	46	Dissection, 20% KOH digestion, FT-IR	67%	Parton et al. (in press)
	*Scyliorhinus stellaris*				
	*Mustelus asterias*				
	*Squalus acanthias*				
	*Scyliorhinus canicula*	20	Dissection, FT-IR	20%	Neves et al.^22^
	*Raja asterias*	7		40%	
North Sea	*Scyliorhinus canicula*	20	Dissection, visual inspection	15%	Smith^36^
**Mediterranean**					
Balearic Islands	*Galeus melastomus*	125	Dissection, FT-IR	17%	Alomar and Deudero^38^
Western Ligurian Sea	*Prionace glauca*	95	Dissection, FT-IR	25%	Bernardini et al.^39^
Tyrrenhenian Sea	*Galeus melastomus*	96	Dissection, 10% KOH digestion, FT-IR	69%	Valente et al.^37^
	*Scyliorhinus canicula*				
	*Etmopterus spinax*				
Ionian Sea	*Pteroplatytrygon violacea*	2	Dissection, Visual inspection	50%	Anastasopoulou et al.^50^
	*Galeus melastomus*	741		3%	
	*Squalus blainville*	75		1%	
	*Etmopterus spinax*	16		6%	
**Pacific**					
Gulf of California	*Rhincodon typus*	12	Skin biopsy (used to infer contaminant levels)	8.42 ng/g w.w. PCBs	Fossi et al.^40^
				1.31 ng/g w.w. DDTs	
				0.29 ng/g w.w. PBDEs	
				0.19 ng/g w.w. HCB	
**Indian**					
KwaZulu-Natal, South Africa	*14 species, see study for details*	15,666	Dissection, Visual inspection	0.38% (macroplastics)	Cliff et al.^62^

Some figures presented here as reported in their respective study.

The contaminants found within our sharks is consistent with other studies investigating the presence of pollutants in the marine environment^23,66–68^, and their colours^23,66,68,69^. Fibres are quickly becoming the most ubiquitous contaminant type in many compartments of marine ecosystems, as well as in the gut contents of numerous marine species including turtles, seals and cetaceans^23,24,70,71^. Fibres have a number of potential sources, including break-off from fishing and maritime equipment such as nets and ropes^72^, fibre shedding from automotive tyre wear and the washing of synthetic fabrics in clothing, as well as breakage and release from other textiles^16,19,73–75^.

We hypothesised that there would be differences in estimated contaminant load among species, between sexes and across size classes. The expected number of ingested fibres was only influenced by individual length (TL cm) with more found in larger sharks. As we were unable to control for location/habitat in this study, this remains to be explored in further detail. While diet could be an additional influencing factor for these shark species, with the current presented data and relatively small sample size we can only speculate as to the factors influencing contaminant burden in these demersal sharks.

### Ingestion pathways

There are at least two potential ingestion pathways for contaminant particles by demersal shark species. Firstly, via the presence of contaminants directly in their food source. Microplastics and other anthropogenic materials have been reported in several prey species for these sharks, including crustaceans and molluscs^28,51,52,76,77^. Some of these prey items have also been to shown to take-up and translocate microplastics around their bodies in laboratory conditions^30,78^, as well as transfer microplastics up the food-web^79^. The species in this study show some variation in their published dietary strategies with starry smooth-hounds and spiny dogfish having fairly specialist diets, compared to small-spotted catsharks and bull huss which are more generalist^44,46,80,81^. We may have expected the generalist feeding sharks to have more contaminants due to feeding on a wider range of prey items, however this was not evident.

The second pathway for exposure could be through direct engulfment alongside target prey species. Habitat use has been identified as a potential driver of plastic ingestion for other elasmobranch species, including whale sharks and manta rays^41^, as well as bony fish species^82^. The sharks analysed in this study all display similar strategies while feeding in their demersal habitat, in that to swallow their prey, they engulf it whole using suction feeding^83,84^. In doing so, many of these species will ingest large quantities of sediment alongside their prey. Although the majority of this is immediately expelled from the mouth, some makes its way to the gut^85,86^. Numerous studies have revealed that microplastics eventually sink to the seafloor and rest in the sediment^87–90^. Naturally occurring sediment particles occur quite regularly in the guts of these shark species and it is highly likely that many of the ingested microfibres will be excreted alongside natural sediment particles. The potential for either plastic or natural particles to cause internal damage before excretion remains to be tested.

Existing studies have attempted to analyse environmental microplastic contamination in the North-East Atlantic, both on the sea surface and the sediment^68,90^. Lusher et al.^68^ found that 94% of samples from surface waters in the North-East Atlantic contained what they believed to be potential microplastics, although after further analysis 63% of these appeared to be matt black anthropogenic fibres and not true microplastics. These matt black fibres are similar in description to many of the fibres found in our current study. When analysed under Raman FT-IR, Lusher et al.^68^ found they were matched closely with cellulose and rayon, again similar to the cellulose fibres found in this study. In a separate study, Maes et al.^90^ identified microplastic particles in 89% of sediment samples from the North Sea and English channel, with most of the plastics considered spheres (microbeads) and fibres^90^, however these authors do not allude to regenerated cellulose fibres in their samples, which may have been present, but not recorded. Given these environmental levels, it should, therefore, be no surprise that approximately 70% of the sharks in this study contained at least 1 contaminant particle.

### Polymer identification

Analysing the polymer make-up of marine plastics can reveal potential sources, fate and causes for ingestion^23,24^. The use of FT-IR spectrometry to analyse environmental samples is a reliable method of determining their polymer make-up^91–93^ and should be fundamental to any future study. The polymers we identified largely reflect the results of similar studies in the marine environment^23,94–96^ and are also similar to polymer diversity of microplastics globally^23,66,97^, with polypropylene being one of the most widely abundant polymers identified worldwide^23^.

Synthetic cellulose fibres are being recorded in environmental samples across multiple studies^23,66,88,98^, although currently their diverse origins remains somewhat understudied. These anthropogenic fibres made up a third of the analysed contaminants and were identified as regenerated cellulose, such as viscose and rayon, as well as lyocell and cotton, with the likely source of such fibres being textiles or personal hygiene items^19,73,74^. Spectral libraries for FT-IR set-ups must continue to expand moving forwards, in order to develop reliable databases that are capable of accurately identifying regenerated cellulose fibres within environmental samples.

Estimates show that an average clothes wash of 6 kg can release more than 700,000 fibres into waste water facilities and although some will be retained by these facilities, many will inevitably make their way into the marine environment, often via river systems^19^. These released fibres such as polyester and cotton are globally in-demand between 24–46 million tonnes per year^99^. Interestingly, the fibre lengths identified in the digestive tracts of sharks were similar to that of fibres released upon washing of various textiles under laboratory conditions (see Fig. 1), highlighting the washing of clothes as a major route for fibres to enter the environment.

### Potential implications

As we only tested a sub-sample of gut content for each animal (20 ml), the proportional incidence of anthropogenic contaminants we report is a conservative estimate. Due to the microscopic size of these synthetic fibres, direct internal organ damage is unlikely, when compared to ingestion of larger macro-plastics, although the ability of small fibres to cause inflammatory damage is acknowledged in other contexts^100–102^. Translocation of relatively large (150 µm) particles can occur across the vertebrate gut via persorption (the passage of particles through the epithelial layers of the gastro-intestinal tract), whilst smaller particles are taken up through normal digestive processes such as pinocytosis and phagocytosis, circulating through the blood and lymph vessels. Thus, there is the opportunity for such circulating particles to enter cells and induce inflammatory damage before being excreted^103^. Fibres of 100–1,000 µm will most likely pass straight through the digestive tract and be excreted with other waste products^23^.

Future research could aim to assess whether certain fibres present exposure risks of associated contaminants and/or persistent organic pollutants^23,24^. Certain textiles may contain toxic chemicals such as BPA (bisphenol A) and BPS (bisphenol S)^104^, with both chemicals capable of causing disruption to reproductive and endocrine systems as well as growth suppression in marine taxa, at relatively low doses^105–108^. Other studies have shown different associated contaminants can present inherent biological risks to various species, including elasmobranchs^34,35,40,109^.

Research has revealed that spiny dogfish and small-spotted catshark are regularly sold in fish and chip shops under pseudonyms such as “Rock”, “Rock salmon” and “Murgey”^110^. If contaminants are able to pass from the digestive tract to the muscle tissue of these shark species, then humans may inadvertently be consuming these pollutants. Although currently there is no conclusive evidence to suggest these pollutants present inherent health risks to humans, we recommend further research to investigate the presence or absence of these contaminant particles in the muscle tissues of these shark species and other fish consumed across the world.

## Conclusions

This study presents the first evidence of microplastics and anthropogenic fibre contaminants in a range of native UK demersal shark species. Although not occurring in as high levels as in other marine megafauna, the presence of anthropogenic particles in these marine species highlights their ubiquitous nature. Although highly unlikely, neither individual nor population level effects of this level of contamination are known. Due to these low levels of ingestion, these species are perhaps not ideal candidates to be used as bio-indicators for marine pollution in demersal habitats when compared to other bony fish species. Nonetheless, if inorganic pollutants can attach to these microfibres, alongside a future increase in their prevalence throughout the marine environment, biological side-effects may occur. Further research on the sources and pathways of anthropogenic fibres may inform policy to reduce their overall prevalence in the environment. By limiting their production in everyday products (through supporting reduction, reuse and replacement of fibre-generating materials from the resource flow) and implementing strategies to prevent their initial entry into the oceans there lies the potential to dramatically reduce the occurrence of microfibres in the marine environment and across food webs.

## Supplementary information

Supplementary Figure S1.

Supplementary Figure S2.

Supplementary Figure S3.

Supplementary Figure S4.

Supplementary Figure S5.

Supplementary Figure S6.

Supplementary Figure S7.

Supplementary Figure S8.

Supplementary Materials.

Supplementary Table S1.

Supplementary Table S2.

## Data Availability

Data is available from the British Oceanographic Centre (Marine).
