# Supplementary figures and images for: Investigating the presence of microplastics in demersal sharks of the North-East Atlantic
Source: Sci Rep. 2020 Jul 22;10:12204. doi: 10.1038/s41598-020-68680-1 (PMC7376218; doi:10.1038/s41598-020-68680-1)

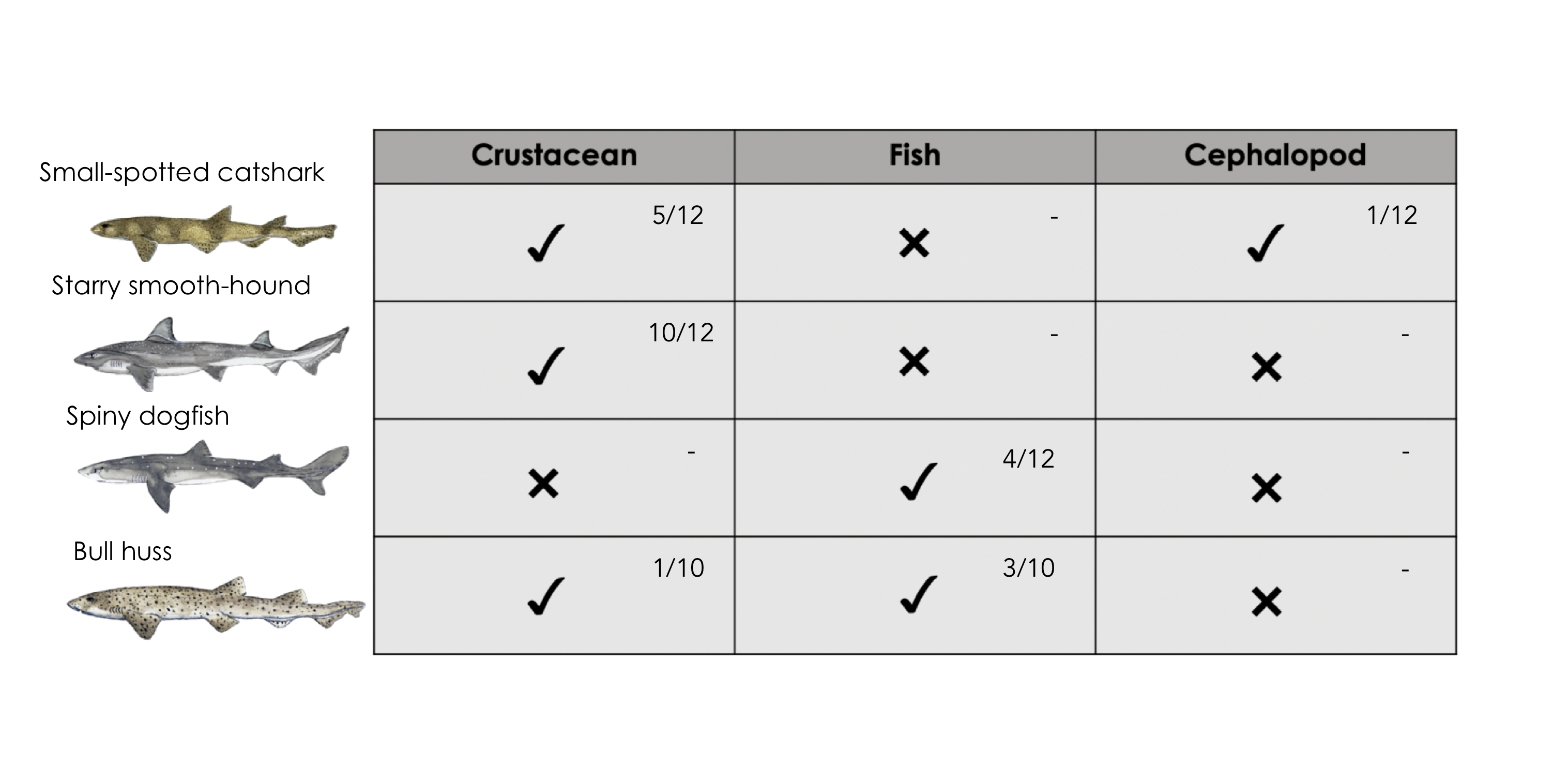

Supplement: Supplementary file 1 — Supplementary Figure S1. [file 41598_2020_68680_MOESM1_ESM.tiff]

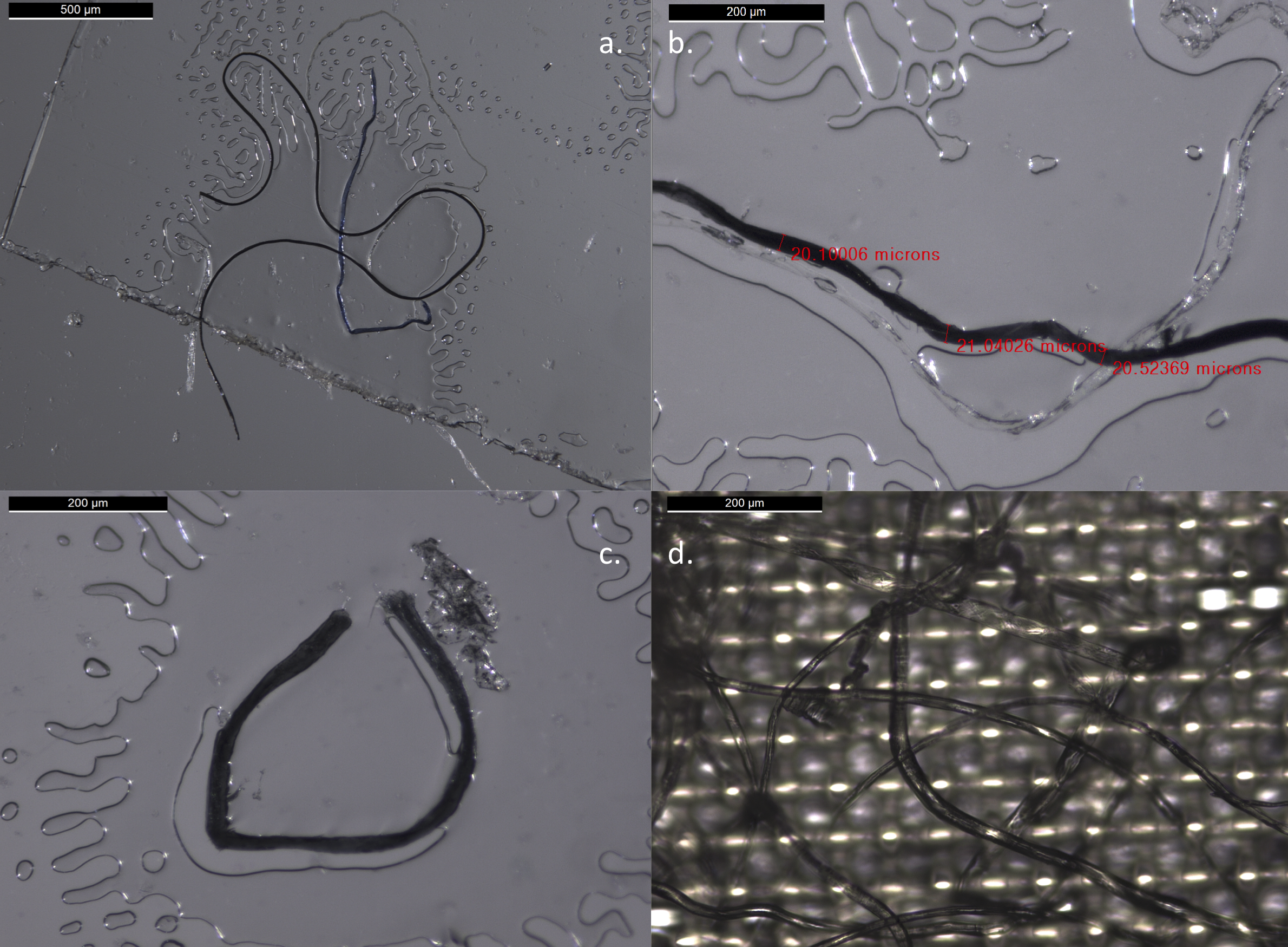

Supplement: Supplementary file 2 — Supplementary Figure S2. [file 41598_2020_68680_MOESM2_ESM.tiff]

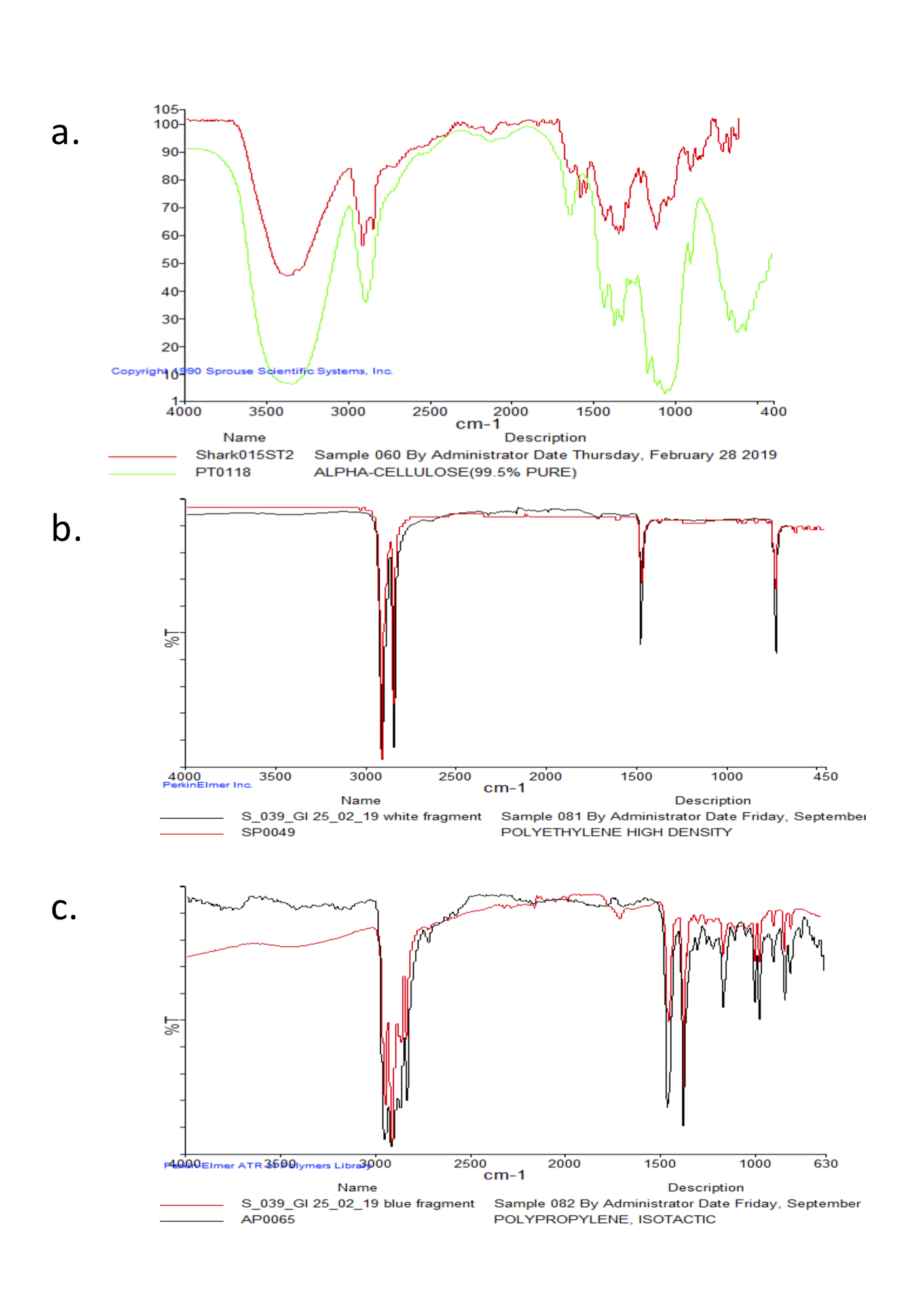

Supplement: Supplementary file 3 — Supplementary Figure S3. [file 41598_2020_68680_MOESM3_ESM.tiff]

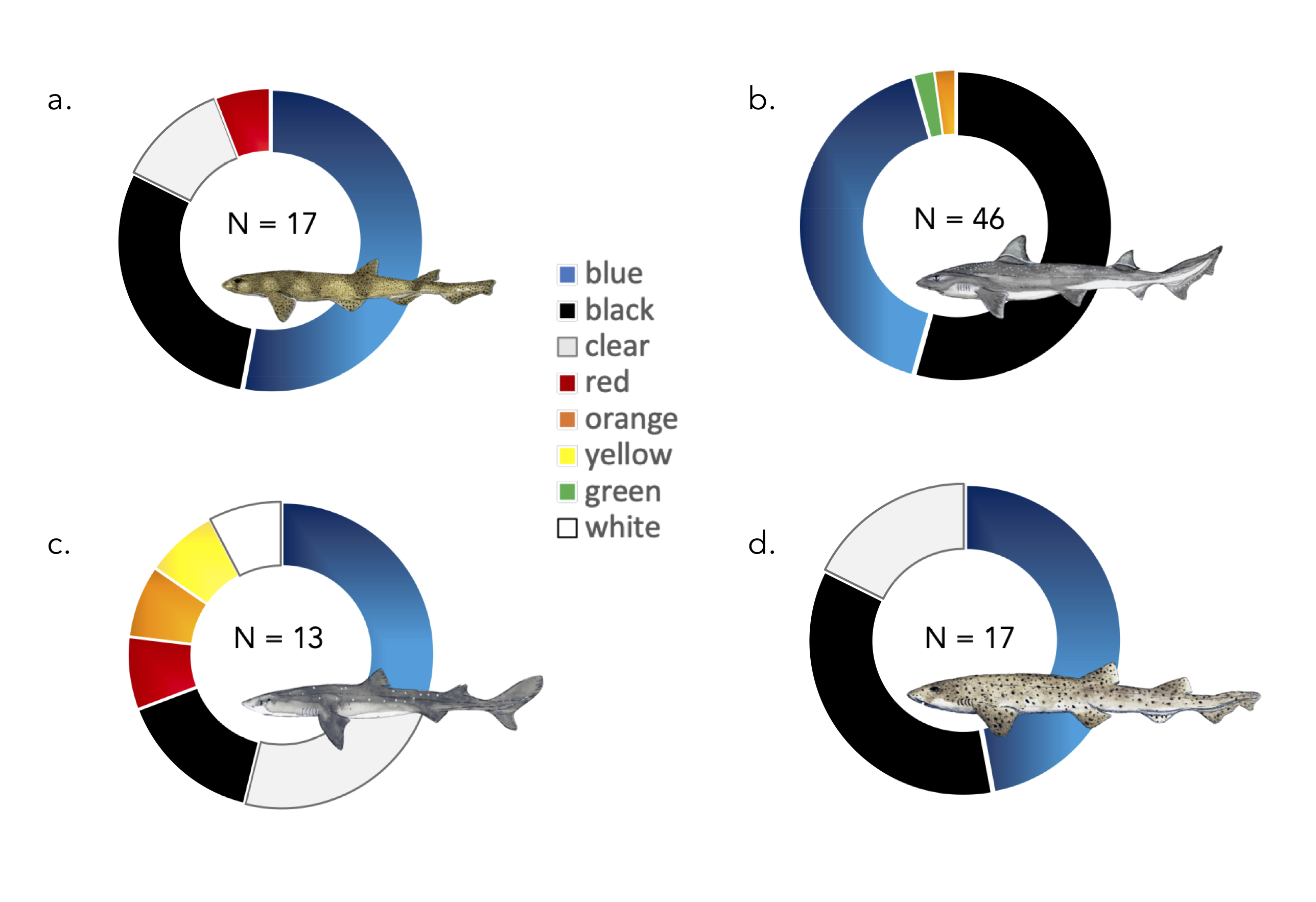

Supplement: Supplementary file 4 — Supplementary Figure S4. [file 41598_2020_68680_MOESM4_ESM.tiff]

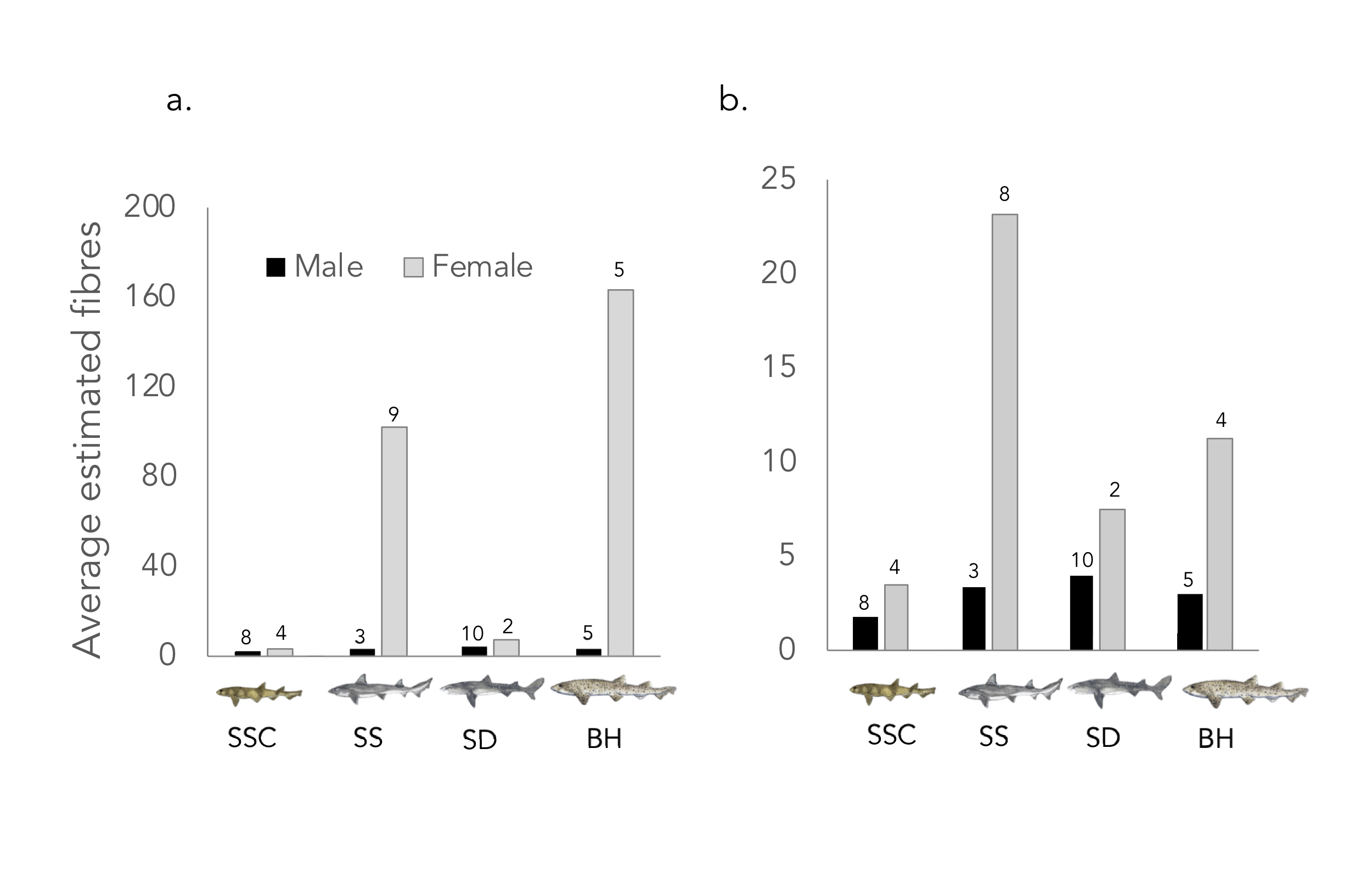

Supplement: Supplementary file 5 — Supplementary Figure S5. [file 41598_2020_68680_MOESM5_ESM.tiff]

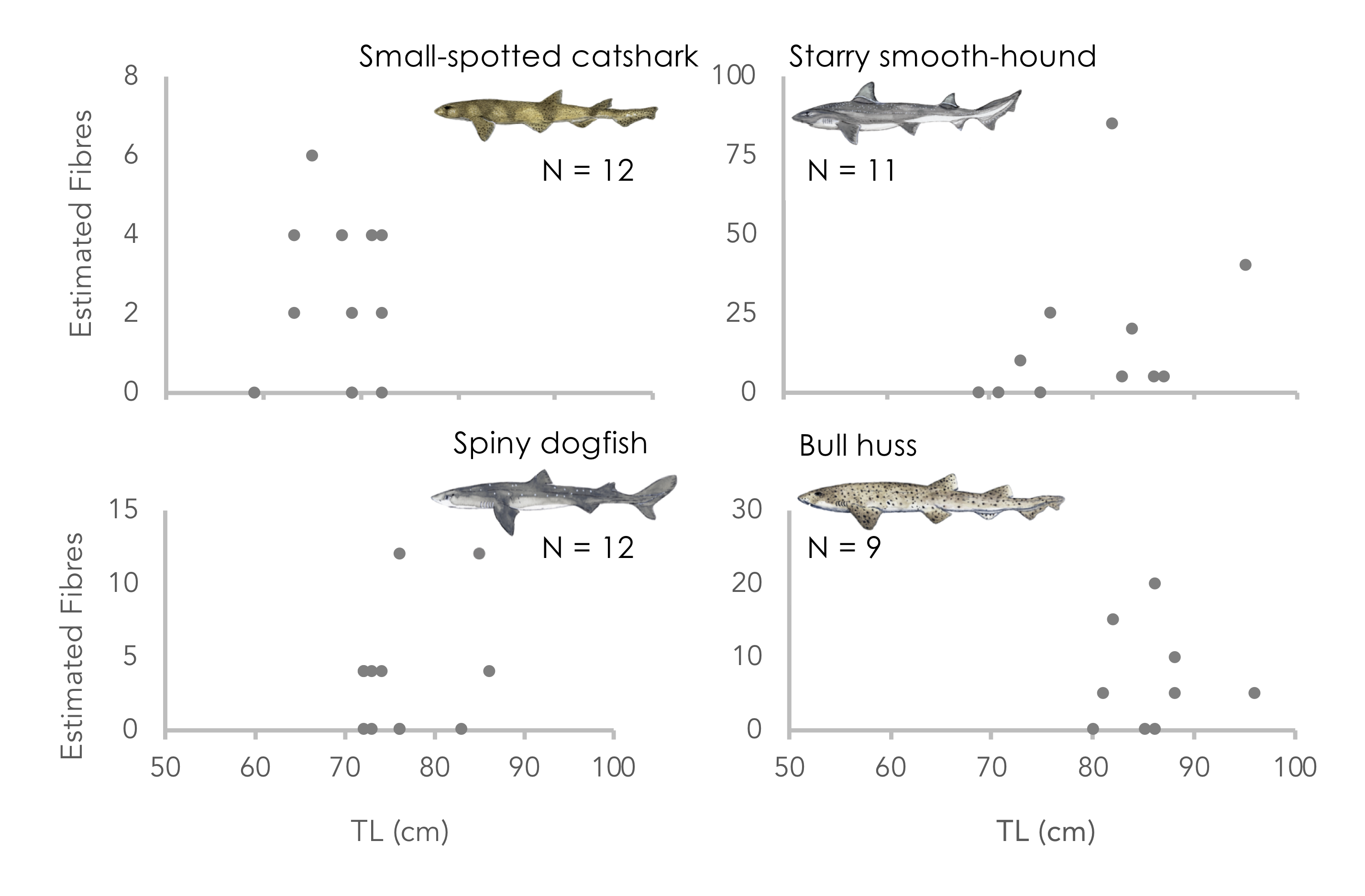

Supplement: Supplementary file 6 — Supplementary Figure S6. [file 41598_2020_68680_MOESM6_ESM.tiff]

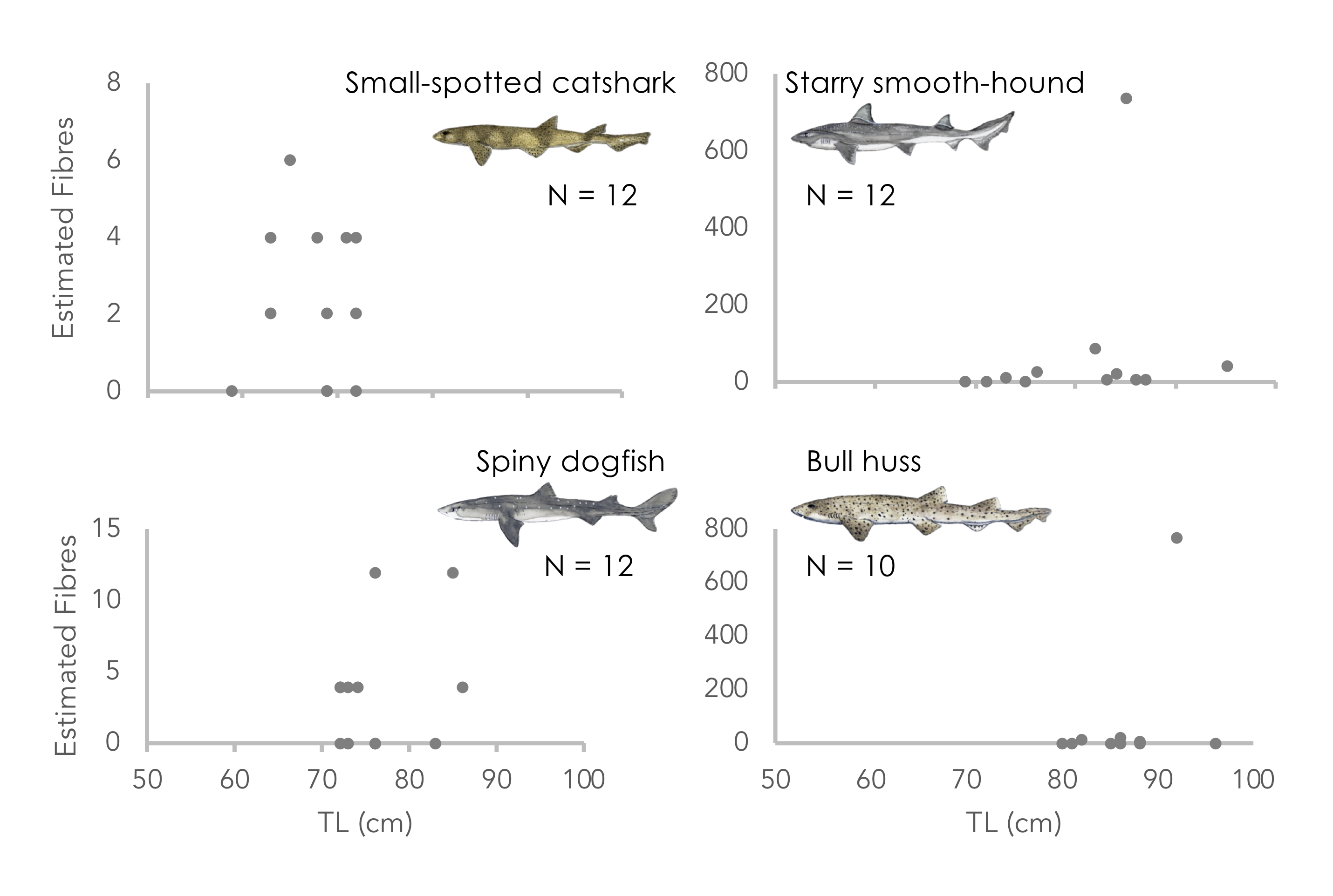

Supplement: Supplementary file 7 — Supplementary Figure S7. [file 41598_2020_68680_MOESM7_ESM.tiff]

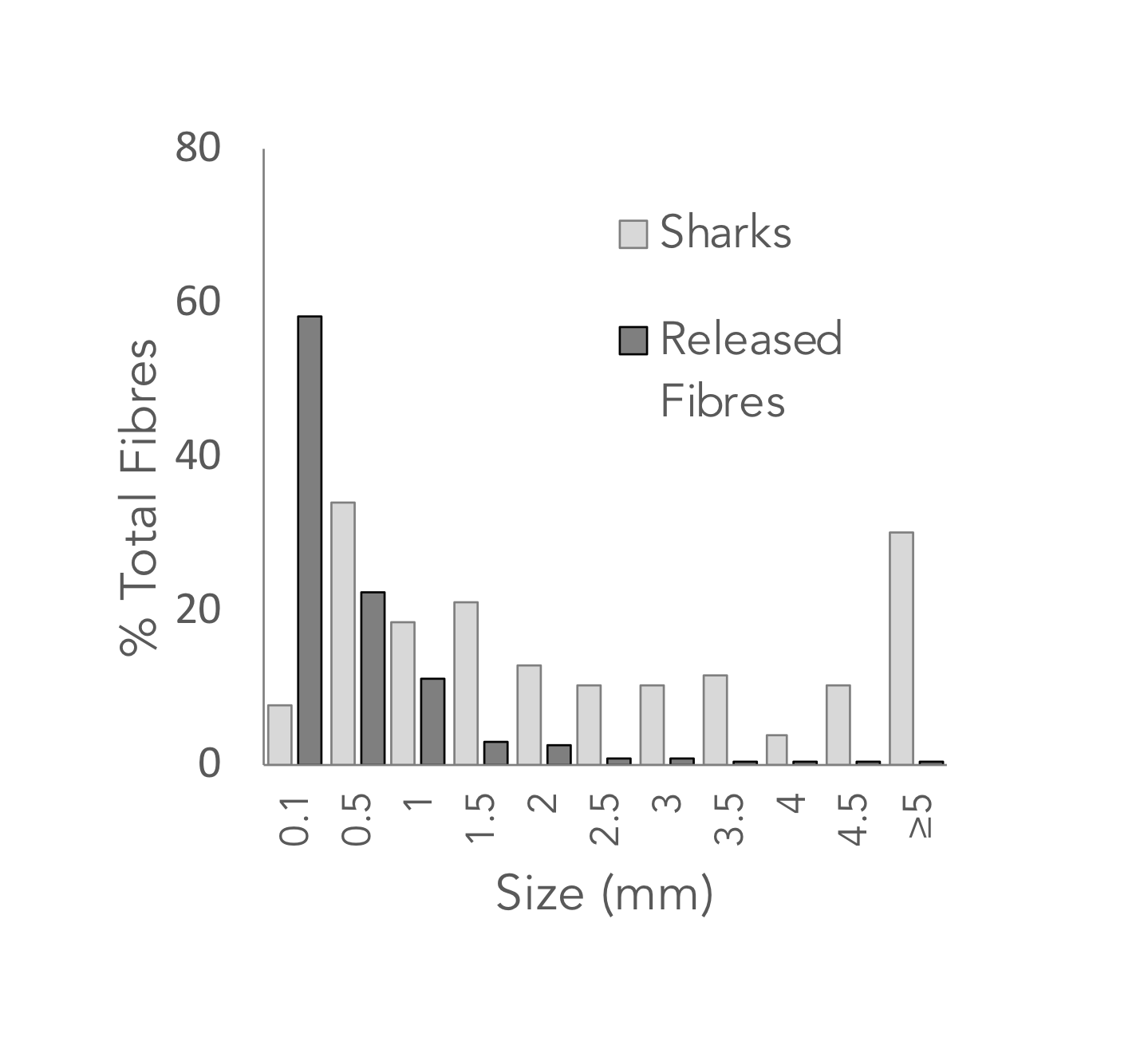

Supplement: Supplementary file 8 — Supplementary Figure S8. [file 41598_2020_68680_MOESM8_ESM.tiff]
