# Supplementary material for: Investigating the presence of microplastics in demersal sharks of the North-East Atlantic
Source: Sci Rep. 2020 Jul 22;10:12204. doi: 10.1038/s41598-020-68680-1 (PMC7376218; doi:10.1038/s41598-020-68680-1)
Supplement: Supplementary file 9 — Supplementary Materials. [file 41598_2020_68680_MOESM9_ESM.pdf]

Supplementary Material

Investigating the presence of microplastics in demersal sharks of the North-East Atlantic

Authors
Kristian J. Parton*[1], Brendan J. Godley[1], David Santillo[2], Muhammad Tausif[3], Lucy C. M. Omeyer[1], Tamara S. Galloway[4]

Addresses
[1] Centre for Ecology & Conservation, College of Life and Environmental Sciences University of Exeter, Penryn Campus TR10 9EZ, United Kingdom
[2]Greenpeace Research Laboratories, School of Biosciences, Innovation Centre Phase 2, University of Exeter, Exeter, United Kingdom
[3] Textile Technology Research Group, School of Design, University of Leeds, LS2 9JT, Leeds, United Kingdom
[4] Biosciences, College of Life and Environmental Sciences, Geoffrey Pope Building, University of Exeter, Stocker Road, Exeter, Devon EX4 4QD, United Kingdom

Supplementary materials

Collection, necropsy and gut content analysis of shark samples

Sharks (n = 46) were obtained from fishermen based down in Cornwall, U.K. All samples were collected and dissected under permission by the University of Exeter ethics committee. Samples of the netting used by the fishermen were also collected and stored for analysis. Four species of NE Atlantic demersal sharks were obtained: small-spotted catshark (Scyliorhinus canicula), spiny dogfish (Squalus acanthias), starry smooth-hound (Mustelus asterias) and bull huss (Scyliorhinus stellaris). Sharks were transported to the University of Exeter, Penryn campus and stored in -80’c freezers until dissection.

Necropsy took place in the post mortem room under sterile conditions. Morphometric shark measurements were taken including: Total length (TL), Precaudal length (PCL), Fork length (FL), First Dorsal height (FDH), Mass (g), Stomach mass (g) and the presence or absence of claspers (M/F). Each species was separated into juvenile or adult individuals based on their size (TL cm) and their genital development.

Upon dissection, the stomach and intestinal tract were removed from the shark species and 10ml of content residue from each was stored in 50ml falcon tubes. This approximated between 20-50% of the total contents of stomach and intestines. Additional notes were taken on the contents of the stomachs to assess what the individuals had been feeding on.

Digestion of samples

Creation of KOH-

20% Potassium hydroxide (KOH-) solution was created using KOH- clusters at a ratio of 200g/1L of filtered water. Filtered water was created using a Nalgene rapid flow filter from filtered water taps in the laboratory. 20% KOH- was added to samples of stomach and intestinal tract at 1:4 ratio using a 40ml glass pipette, washed with Milli-Q water between uses. Treated samples were later oven heated for 48 hours at 60’C to aid in the digestion process.

Filtering of Samples

Filtered water was initially run through a Millipore filtration kit (MFK) to remove any contaminants present on the equipment, this was repeated between each sample. Treated samples were shaken and subsequently run through the MFK onto 30um filter paper cut into 6cm diameter circles. Biological material retained on the inside of the filtration kit was flushed through the filtration kit with Milli-Q water. Upon filtration, the 30um filters were quickly removed using stainless steel tweezers and placed into petri dishes, which were subsequently sealed with masking tape and stored for later analysis.

Microscopy analysis

Filtered shark samples were examined under a digital stereo light microscope (Leica M165C) at 8x magnification and scanned for contaminants. Samples were scanned across horizontally until all of the sample had been viewed. Microplastic contaminants were recorded and categorised as either: fibres, beads or fragments and further subcategorised into 5 colour categories: red, blue, black, yellow or other. Length of contaminants were measured, alongside the smallest diameter of any suspected fragments and beads and photographed by a digital camera (Leica DFC295; Leica Suite Application Version 3.6.X).

Contamination prevention

Personal protective equipment was used at all times. As some microplastics/fibres may be on clothing, attached to laboratory equipment or airborne, we undertook several steps to control for and prevent contamination of shark samples. All equipment and apparatus were rinsed thoroughly throughout with Milli-Q water as well as between uses. Surfaces were wiped down with 70% ethanol prior to work commencing. Airborne contamination blanks (N = 25, one per bout of laboratory work) consisting of filter paper dampened with filtered water placed in a petri dish) were run throughout all stages of the process and were sealed with masking tape and stored for microscopic analysis upon completion of dissections, oven-heating, filtrations and microscopic analyses. Analysis of these filters showed minimal evidence of contamination with the presence of some fibres (n = 6 cases of single

fibres), that visually appeared different to those found in the shark samples. As an extra precaution, for any samples processed during the same bout, if they contained any fibres of the same colour these were discounted.

Procedural blanks (N = 24) were treated in the same way as the shark gut content samples and were run parallel to the digestion, oven-heating and filtration processes. These were poured through the 30um mesh filters (as per the methods) and were stored for microscopic analysis to check for contamination. No evidence of any microplastic contamination was found.

Polymer Identification

A subsample of contaminants (n = 62) were investigated using Fourier Transform Infrared spectroscopy (FT-IR) to determine their polymer make-up.

Individual candidate materials (fibres and fragments) were positioned on the surface of a silver filter (47 mm diameter silver-coated membrane filter, pore size 5 μm, Sterlitech) held in a glass petri dish and their positions marked by scratching the filter surface both to facilitate orientation under the microscope and to ensure that only those fibres and fragments originating from the samples were subsequently analysed (i.e. to avoid any possible interference from airborne microplastics). Both the silver filters and petri dishes had been inspected before use using a dissecting stereomicroscope under both low and high magnification in order to verify that they were completely free from fibres and fragments. Candidate materials were examined using a PerkinElmer Spotlight 400 FT-IR Imaging System (MCT detector, KBr window) operating in reflectance mode across a wavenumber range from 4000 to 750 cm-1 and with a resolution of 4 cm-1.

The infrared spectra were acquired, processed and analysed using PerkinElmer Spectrum software (version 10.5.4.738), with polymers being identified by automated matching combined with expert judgment against commercially available spectral libraries (including polymers, additives, solvents, etc.) and an additional custom spectral library prepared in our laboratory using a range of polymer standards and potential contaminating materials (e.g. tissues, gloves, laboratory coats).

Any fibres or fragments appearing on the filters other than those previously marked were excluded.The comparisons were made using PerkinElmer Spectrum software (version 10.5.4.738), incorporating a total of 8 different commercially available spectral libraries relating to polymers, polymer additives and adhesives as provided by PerkinElmer (adhes.dlb, Atrpolym.dlb, ATRSPE~1.DLB, fibres.dlb, IntPoly.spl, poly1.dlb, polyadd1.dlb & POLYMER.DLB) as well as an additional library compiled at the Greenpeace Research Laboratories in order to exclude common laboratory contaminants (fibres from tissues, blue roll, laboratory coats, glove fragments, etc.). The Spectrum software allows for the simultaneous comparison of spectra obtained for a sample against all nine libraries, and reports the 10 most likely matches across all of those libraries, in each case, matches which were then subsequently checked by the analyst in order to verify the quality of the match and the reliability of the identification.

On samples where there were multiple contaminants, a minimum of 5 contaminants were selected for analysis with FT-IR. Scores greater than 65% were considered reliable spectral matches. Some spectral matches of cellulose fibres between 65-70% were sent for visual analysis at Leeds university to confirm their identity by light microscopy / image analysis and were eventually accepted.

Statistical Analysis

A negative binomial generalised linear model (GLM) was used to investigate the influence of species, sex, and individual length on the expected number of ingested fibres, using the MASS package[1] in R v3.5.1.[2] All combinations of terms were examined and ranked by Akaike's Information Criteria (AIC) using subset selection of the maximal model using the MuMIn package v1.42.1.[3] Top ranked models were defined as models ΔAIC ≤ 2 units of the best supported model, after excluding further models where a simpler model attained stronger weighting[4].

Supplementary Figures

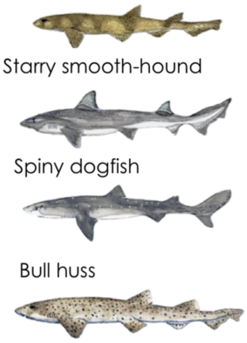

	Crustacean	Fish	Cephalopod
Small-spotted catshark	✓ 5/12	✗ -	✓ 1/12
Starry smooth-hound	✓ 10/12	✗ -	✗ -
Spiny dogfish	✗ -	✓ 4/12	✗ -
Bull huss	✓ 1/10	✓ 3/10	✗ -

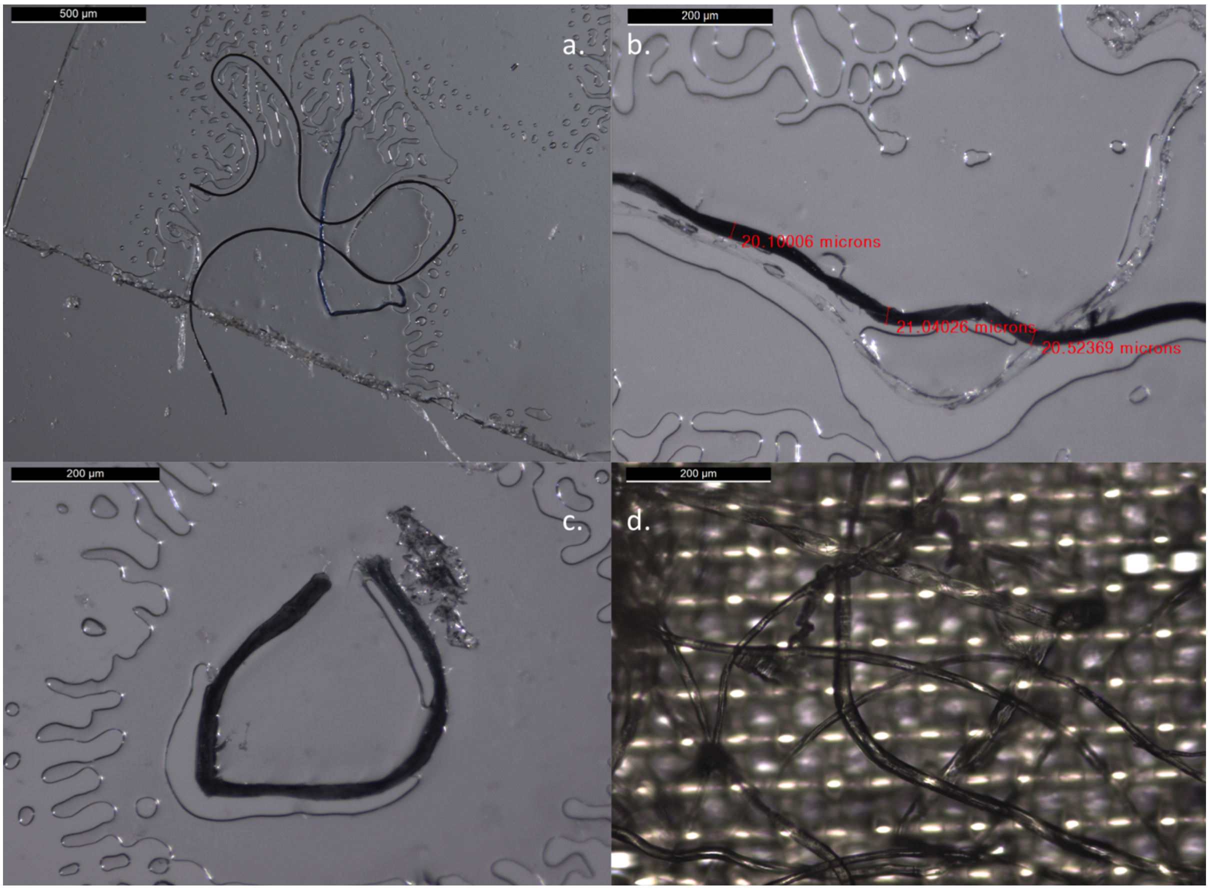
500 µm
a.
200 µm
b.
20.10006 microns
21.04026 microns
20.52369 microns
200 µm
c.
200 µm
d.

a.

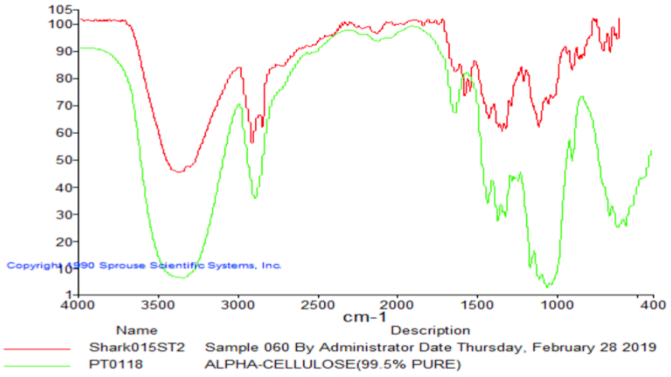
105
100
90
80
70
60
50
40
30
20
10
1
4000
3500
3000
2500
2000
1500
1000
400
cm-1
Copyright 1990 Sprouse Scientific Systems, Inc.
Name
Description
Shark015ST2
Sample 060 By Administrator Date Thursday, February 28 2019
PT0118
ALPHA-CELLULOSE(99.5% PURE)

b.

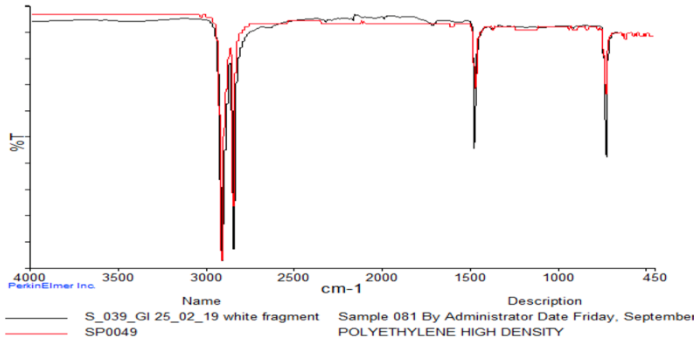
%T
4000
3500
3000
2500
2000
1500
1000
450
cm-1
PerkinElmer Inc.
Name
Description
S_039_GI 25_02_19 white fragment
Sample 081 By Administrator Date Friday, September
SP0049
POLYETHYLENE HIGH DENSITY

c.

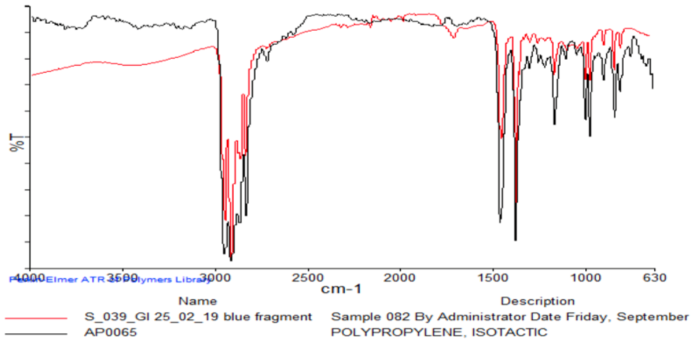
%T
4000
3500
3000
2500
2000
1500
1000
630
cm-1
Name
Description
S_039_GI 25_02_19 blue fragment
Sample 082 By Administrator Date Friday, September
AP0065
POLYPROPYLENE, ISOTACTIC

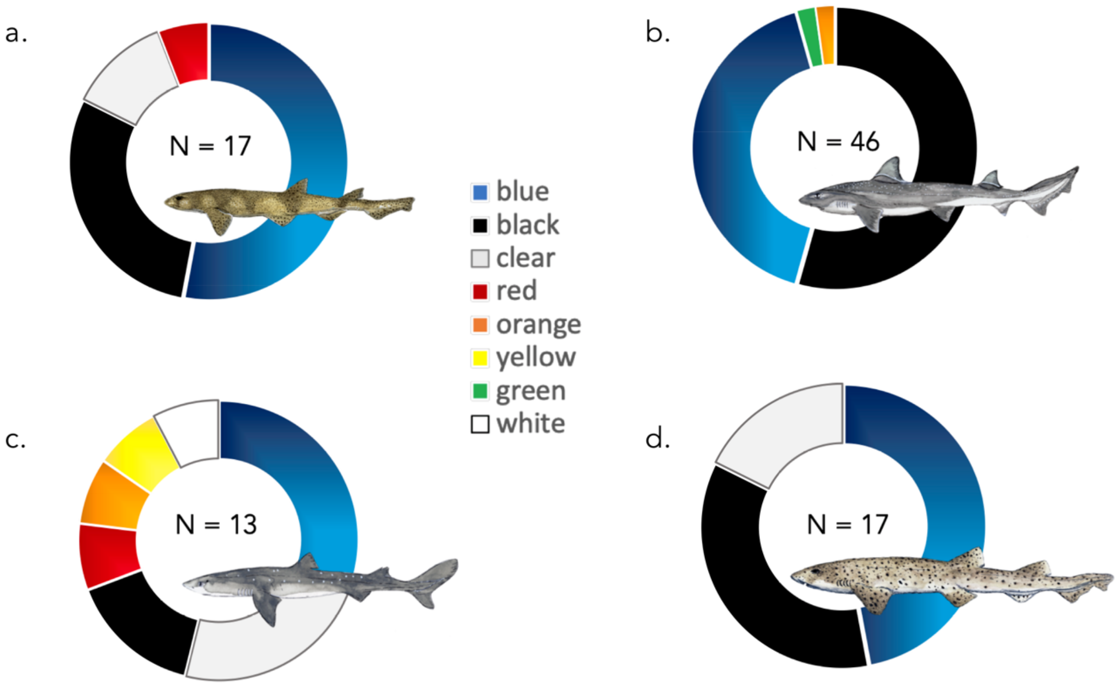
a.
N = 17
b.
N = 46
c.
N = 13
d.
N = 17
blue
black
clear
red
orange
yellow
green
white

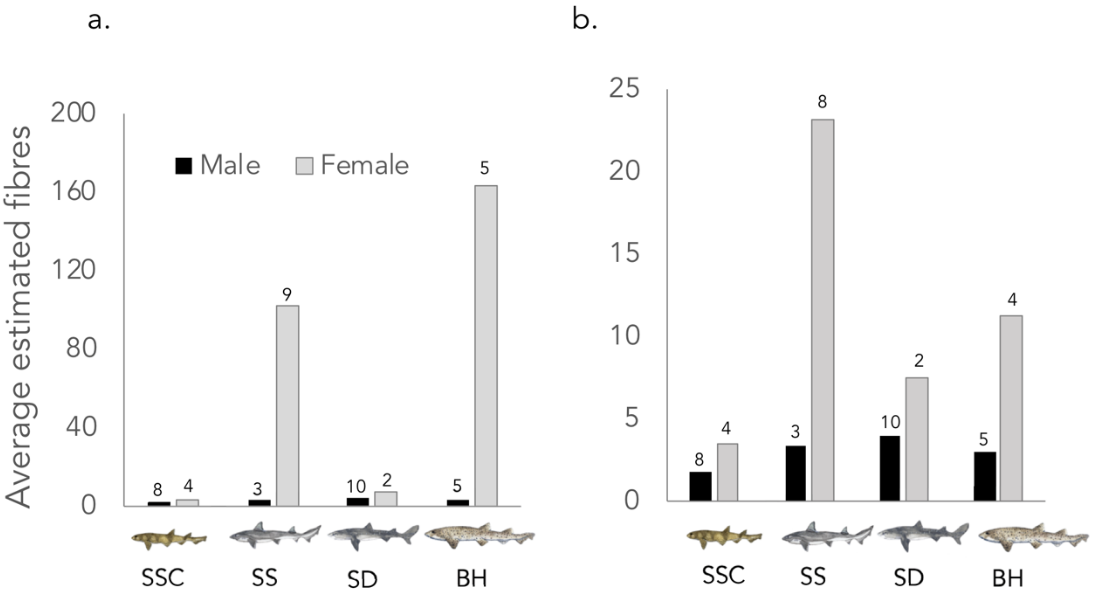

a.
b.
Average estimated fibres
Male
Female
200
160
120
80
40
0
25
20
15
10
5
SSC
SS
SD
BH

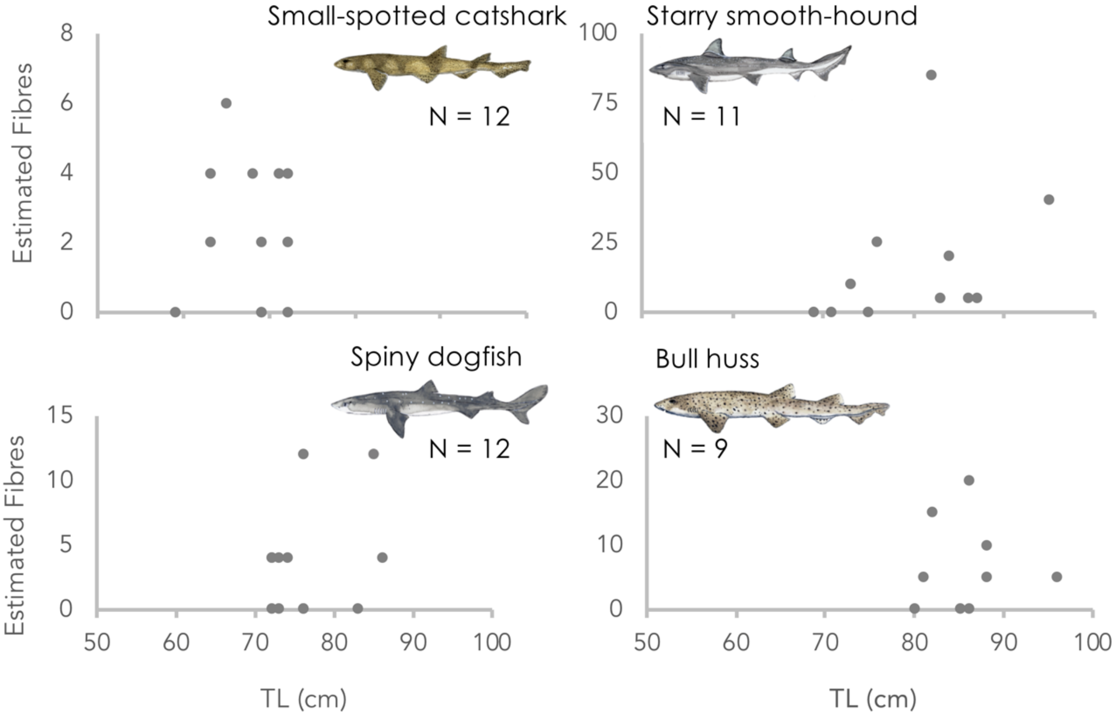

Small-spotted catshark
N = 12
Starry smooth-hound
N = 11
Spiny dogfish
N = 12
Bull huss
N = 9
Estimated Fibres
Estimated Fibres
TL (cm)
TL (cm)

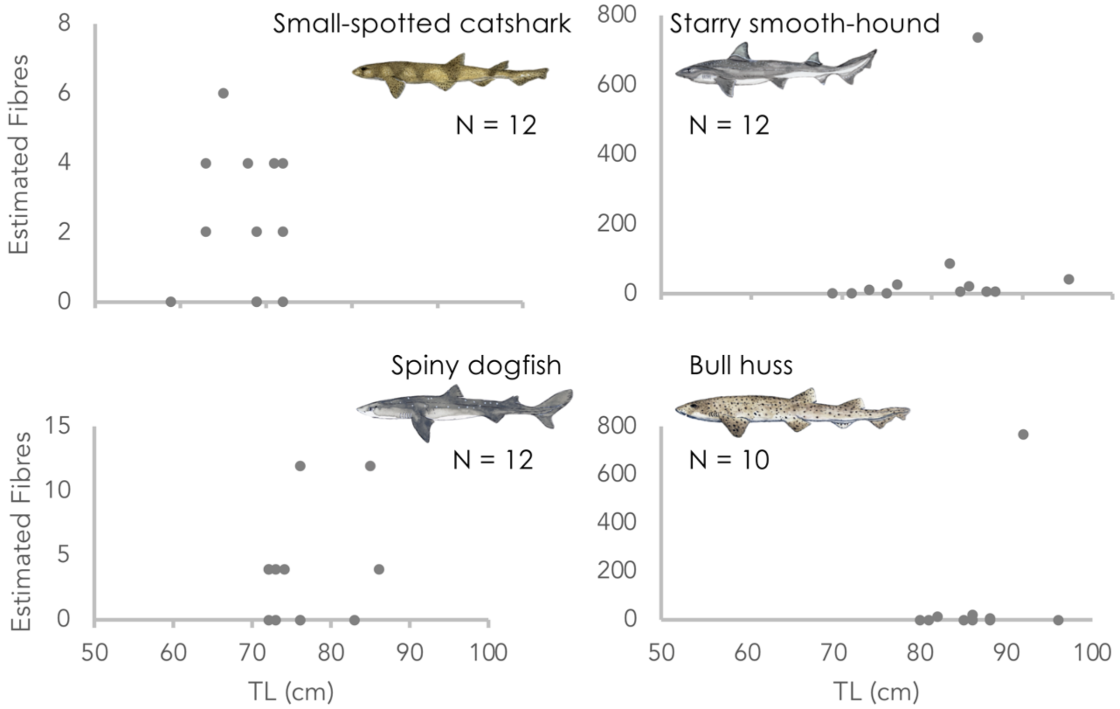

Small-spotted catshark
N = 12
Starry smooth-hound
N = 12
Spiny dogfish
N = 12
Bull huss
N = 10
Estimated Fibres
TL (cm)
8
6
4
2
0
800
600
400
200
0
15
10
5
0
800
600
400
200
0
50
60
70
80
90
100

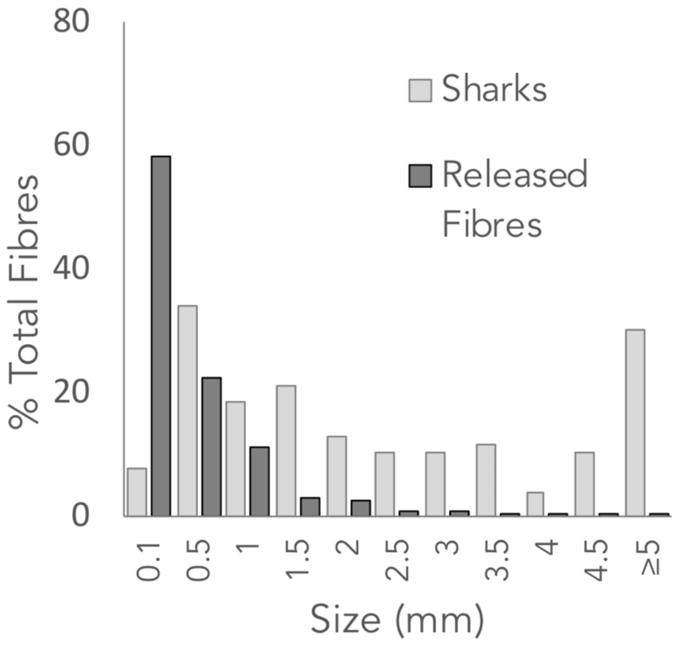

80
60
40
20
0
% Total Fibres
Sharks
Released Fibres
0.1
0.5
1
1.5
2
2.5
3
3.5
4
4.5
≥5
Size (mm)

\

Supp Table S1: Results from the subsample of isolated particles (N = 62) analysed using Fourier transform infrared spectroscopy (FT-IR) to determine their polymer make up from gut content residue samples of UK demersal sharks. SSC: small spotted catshark, SS: starry smooth-hound, SD: spiny dogfish, BH: bull huss. Percentage of synthetic contaminants annotated in table.

Origin	Group	FT-IR Identification	SSC	SS	SD	BH
**Synthetics**	Plastics	Olefin Polypropylene fibres	-	5	-	10
		Polypropylene fragment	-	-	-	1
		Polyacrylamide	5	-	1	-
		Polyester fibres	-	3	1	1
		Polyethylene fragment	-	-	1	-
	Regenerated Cellulose	Rayon or Viscose	8	7	1	4
		Cellophane	-	1	-	-
			**86.6%**	**84.2%**	**57.1%**	**76.2%**
**Non-synthetics / Low spectral match scores**	Other	Hexocyclium	1	-	-	-
		Thiobis	1	-	-	-
		Acetyl triethyl citrate	-	1	-	-
		Ethylene (Low match score)	-	1	-	-
		Poly(film) (Low match score)	-	1	-	-
		Ethyl cellulose (Low match score)	-	-	1	-
		D-biotin	-	-	2	-
		Polyacrylonitrile (Low match score)	-	-	-	1
		Erthryose	-	-	-	1
		Cyanide (Low match score)	-	-	-	1
		Mercuric (Low match score)	-	-	-	1
		Human umbilical cords (Low match score)	-	-	-	1
**Total:**			**15**	**19**	**7**	**21**

Supp Table S2: Summary results of negative binomial generalised linear model. Top ranked model and adjusted weight after selection for ∆AIC ≤ 2 and applying the nesting rule. Top set model highlighted in bold.

Response variable	Fixed effects	Intercept	d.f.	logLik	AIC	∆AIC	Weight	Adj. weight
**Expected fibres**	**~ Length**	**-11.57**	**3**	**-149.26**	**304.5**	**0.00**	**0.37**	**1.00**
	~ 1	3.685	2	-160.62	325.2	20.72	0.00	

d.f.: degrees of freedom. logLik: log likelihood. AIC: Akaike's Information Criterion. Adj. weight: adjusted weight.

References

1. Venables WN, Ripley BD (2002) Modern applied statistics with S (fourth edition). Springer, New York (USA)
2. R Core Team (2018) R: A language and environment for statistical computing. R Found Stat Comput Vienna, Austria
3. Barton K (2015) MuMIn: Multi-model inference. https:// CRAN.R-project.org/package=MuMIn.
4. Richards SA, Whittingham MJ, Stephens PA (2011) Model selection and model averaging in behavioural ecology: The utility of the IT-AIC framework. Behav Ecol Sociobiol 65:77–89

Figure Legends:

1. **Supp Figure S1:** Dietary tick chart. Different dietary items found/not found in each species during visual inspection of stomach contents. Frequency occurrence annotated on figure. "-" = Not found. Some contents were too digested to visually determine their origins and therefore are not included in the counts here. Elasmobranch drawings by Lucie Jones.

2. **Supp Figure S2:** Microscope imagery of fibres found in shark samples, as well as laboratory treated known fibre types. a.) Cellulosic fibre - 500um scale bar. b.) Cellulosic fibre - 200um scale bar, with added measurements displaying uniform diameter indicative of anthropogenic fibres. c.) Cellulosic fibre, 200um scale bar, displaying damaged fibre end. d.) Laboratory treated cotton fibres, 200um scale bar, showing dimensional and morphological similarities to fibres found within shark samples.

3. **Supp Figure S3:** FT-IR spectra. a.) Spectra for cellulosic fibres presumed to be cotton/regenerated cellulose. b. Spectra for polyethylene fragment found in shark sample. c.) Spectra for polypropylene fragment found in shark sample.

4. **Supp Figure S4:** Fibre colour composition with extreme values removed. Pie charts representing colours of ingested fibres, found across both the stomachs and intestines of four species of north-east atlantic demersal sharks: a. small-spotted catshark (*Scyliorhinus canicula*), b. starry smooth-hound (*Mustelus asterias*), c. spiny dogfish (*Squalus acanthias*) and d. bull huss (*Scyliorhinus stellaris*). Total N of coloured fibres identified annotated within figure. Elasmobranch drawings by Lucie Jones.

5. **Supp Figure S5:** Average estimated fibres breakdown between males and females. a. Two extreme values included (one female starry smooth-hound & one female bull huss). b. Two extreme values removed. SSC: small-spotted catshark, SS: starry smooth-hound, SD: spiny dogfish, BH: bull huss. N of Males/Females sampled annotated above bar. Elasmobranch drawings by Lucie Jones.

6. **Supp Figure S6:** Estimated fibres as a function of total length (TL cm) for four shark species. N = annotated. Two extreme values removed (one starry smooth-hound, TL: 85cm, estimated fibres: 735, one bull huss, TL: 92cm, estimated fibres: 770). Elasmobranch drawings by Lucie Jones.

7. **Supp Figure S7:** Estimated fibres as a function of total length (TL cm) for four shark species. N = annotated. Extreme values included. Elasmobranch drawings by Lucie Jones.

8. **Supp Figure S8:** Fibre length distribution with extreme values removed from shark data. Fibre lengths as a proportion of total fibres for fibres found in shark species (light grey) and fibres released in laboratory conditions after washing of various cotton and polyethylene terephthalate textiles. Palacios Marin AV, (2019) Release of microfibres from comparative common textile structures during laundering (Unpublished Masters dissertation). University of Leeds, UK.

9. **Supp Table S1**: Results from the subsample of isolated particles (N = 62) analysed using Fourier transform infrared spectroscopy (FT-IR) to determine their polymer make up from gut content residue samples of UK demersal sharks. SSC: small spotted catshark, SS: starry smooth-hound, SD: spiny dogfish, BH: bull huss. Percentage of synthetic contaminants annotated in table.

10. **Supp Table S2**: Summary results of negative binomial generalised linear model. Top ranked model and adjusted weight after selection for $\Delta AIC \leq 2$ and applying the nesting rule. Top set model highlighted in bold.
