# Supplementary material for: Investigating the presence of microplastics in demersal sharks of the North-East Atlantic
Source: Sci Rep. 2020 Jul 22;10:12204. doi: 10.1038/s41598-020-68680-1 (PMC7376218; doi:10.1038/s41598-020-68680-1)
Supplement: Supplementary file 10 — Supplementary Table S1. [file 41598_2020_68680_MOESM10_ESM.docx]

| **Origin** | **Group** | **FT-IR Identification** | **SSC** | **SS** | **SD** | **BH** |
| --- | --- | --- | --- | --- | --- | --- |
| **Synthetics** | Plastics | Olefin Polypropylene fibres  Polypropylene fragment  Polyacrylamide  Polyester fibres  Polyethylene fragment | -  -  5  -  - | 5  -  -  3  - | -  -  1  1  1 | 10  1  -  1  - |
|  | Regenerated Cellulose | Rayon or Viscose  Cellophane | 8  - | 7  1 | 1  - | 4  - |
|  |  |  | **86.6%** | **84.2%** | **57.1%** | **76.2%** |
| **Non-synthetics / Low spectral match scores** | Other | Hexocyclium  Thiobis  Acetyl triethyl citrate  Ethylene (Low match score)  Poly(film) (Low match score)  Ethyl cellulose (Low match score)  D-biotin  Polyacrylonitrile (Low match score)  Erthryose  Cyanide (Low match score)  Mercuric (Low match score)  Human umbilical cords (Low match score) | 1  1  -  -  -  -  -  -  -  -  -  - | -  -  1  1  1  -  -  -  -  -  -  - | -  -  -  -  -  1  2  -  -  -  -  - | -  -  -  -  -  -  -  1  1  1  1  1 |
|  |  |  |  |  |  |  |
| **Total:** |  |  | **15** | **19** | **7** | **21** |

**Supp Table S1**: Results from the subsample of isolated particles (N = 62) analysed using Fourier transform infrared spectroscopy (FT-IR) to determine their polymer make up from gut content residue samples of UK demersal sharks. SSC: small spotted catshark, SS: starry smooth-hound, SD: spiny dogfish, BH: bull huss. Percentage of synthetic contaminants annotated in table.
