# Supplementary material for: Investigating the presence of microplastics in demersal sharks of the North-East Atlantic
Source: Sci Rep. 2020 Jul 22;10:12204. doi: 10.1038/s41598-020-68680-1 (PMC7376218; doi:10.1038/s41598-020-68680-1)
Supplement: Supplementary file 11 — Supplementary Table S2. [file 41598_2020_68680_MOESM11_ESM.docx]

| Response variable | Fixed effects | Intercept | d.f. | logLik | AIC | ΔAIC | Weight | Adj. weight |
| --- | --- | --- | --- | --- | --- | --- | --- | --- |
| **Expected fibres** | **~ Length** | **-11.57** | **3** | **-149.26** | **304.5** | **0.00** | **0.37** | **1.00** |
|  | ~ 1 | 3.685 | 2 | -160.62 | 325.2 | 20.72 | 0.00 |  |

**Supp Table S2**: Summary results of negative binomial generalised linear model. Top ranked model and adjusted weight after selection for ΔAIC ≤ 2 and applying the nesting rule. Top set model highlighted in bold.

d.f.: degrees of freedom. logLik: log likelihood. AIC: Akaike’s Information Criterion. Adj. weight: adjusted weight.
